# Effects of individualized PEEP obtained by two different titration methods on postoperative atelectasis in obese patients: study protocol for a randomized controlled trial

**DOI:** 10.1186/s13063-021-05671-1

**Published:** 2021-10-15

**Authors:** Qing-Yuan Wang, Yu-Wei Ji, Li-Xin An, Lei Cao, Fu-Shan Xue

**Affiliations:** Department of Anesthesiology, Beijing Friendship Hospital, Capital Medical University, No. 95 Yongan Road, Xicheng District, Beijing, 100050 China

**Keywords:** Obesity, Individualized PEEP, Cstat, Electrical impedance tomography, Atelectasis

## Abstract

**Background:**

The incidence of postoperative pulmonary complications (PPCs) is higher in obese patients undergoing general anesthesia and mechanical ventilation due to the reduction of oxygen reserve, functional residual capacity, and lung compliance. Individualized positive end-expiratory pressure (iPEEP) along with other lung-protective strategies is effective in alleviating postoperative atelectasis. Here, we compared the best static lung compliance (Cstat) titration of iPEEP with electrical impedance tomography (EIT) titration to observe their effects on postoperative atelectasis in obese patients undergoing laparoscopic surgery.

**Methods:**

A total number of 140 obese patients with BMI ≥ 32.5kg/m^2^ undergoing elective laparoscopic gastric volume reduction and at moderate to high risk of developing PPCs will be enrolled and randomized into the optimal static lung compliance-directed iPEEP group and EIT titration iPEEP group. The primary endpoint will be pulmonary atelectasis measured and calculated by EIT immediately after extubation and 2 h after surgery. Secondary endpoints will be intraoperative oxygenation index, organ dysfunction, incidence of PPCs, hospital expenses, and length of hospital stay.

**Discussion:**

Many iPEEP titration methods effective for normal weight patients may not be appropriate for obese patients. Although EIT-guided iPEEP titration is effective in obese patients, its high price and complexity limit its application in many clinical facilities. This trial will test the efficacy of iPEEP via the optimal static lung compliance-guided titration procedure by comparing it with EIT-guided PEEP titration. The results of this trial will provide a feasible and convenient method for anesthesiologists to set individualized PEEP for obese patients during laparoscopic surgery.

**Trial registration:**

ClinicalTrials.govChiCTR2000039144. Registered on October 19, 2020

**Supplementary Information:**

The online version contains supplementary material available at 10.1186/s13063-021-05671-1.

## Background

The incidence of postoperative pulmonary complications (PPCs) in obese patients is as high as 18%, which is almost twice that in normal weight or overweight patients [1, 2]. It is widely acknowledged that PPCs cause a significant increase in morbidity and mortality, prolonged length of hospital stay and ICU stay, and increased treatment expenses [2, 3]. Considering that roughly 310 million patients undergo surgery worldwide annually [4], reducing the rate of PPCs should have a positive impact on mortality and morbidity. It could also improve patients’ prognosis and reduce health system expenses.

According to an international expert consensus [5], lung-protective ventilation strategies including low tidal volume [6], recruitment maneuvers, individualized PEEP, and the lowest possible oxygen concentration have been associated with reduced incidence of postoperative pulmonary complications. Among these, individualized PEEP plays an important role in preventing processive alveolar collapse; however, no agreement has been reached about how to select individualized PEEP.

Patients whose BMI equal to or exceed 35 kg/m^2^ are more susceptible to developing PPCs compared with mild obese and normal weight patients. In obese patients, respiratory function is undermined due to the decreased oxygen reserve, respiratory compliance, and functional residual capacity [7]. Moreover, obese patients are prone to develop atelectasis, which is further exacerbated by general anesthesia and mechanical ventilation. When obese patients undergo laparoscopic surgery, the cephalic shift of diaphragm and elevated pleural pressure imposed by pneumoperitoneum increase the proportion of atelectatic lung tissue. An intraoperative higher level of PEEP with alveolar recruitment maneuvers may improve respiratory function in obese patients [7, 8].

Recently, PROBESE trial, an international randomized trial, using a higher level of PEEP (12 cmH_2_O) and ARM compared with a lower level of PEEP (4 cmH_2_O) in obese patients, did not reduce PPCs [1, 9]. The conclusion of this study was not consistent with previous studies [7, 8]. However, the lack of individual titration of PEEP is their major limitation [1]. Nestler and his colleagues [8] revealed that in obese patients undergoing elective laparoscopic surgery, compared to patients ventilated with a fixed PEEP of 5 cmH_2_O, patients ventilated with individualized PEEP titrated through an electrical impedance tomography (EIT)-guided procedure experience alleviated atelectasis, improved regional ventilation distribution and oxygenation, and decreased driving pressure. Therefore, for obese patients, the appropriate individual PEEP rather than a single high level of PEEP may be more beneficial. However, the EIT instrument is expensive, making it unavailable for many medical institutions. In some thoracic and abdominal operations, the scope of disinfection also limits its application. The complex techniques related to EIT further impair its feasibility in routine clinical practice. Therefore, some simple and highly feasible methods to titrate individualized PEEP for obese patients need to be found out.

This trial is going to verify the hypothesis: individualized PEEP titrated by optimal static lung compliance (Cstat) in combination with other lung-protective ventilation strategies is not inferior to individualized PEEP titrated via EIT in terms of reducing atelectasis, improving oxygenation and reducing the incidence of PPCs in obese patients at moderate to high risk of developing PPCs.

## Methods/design

### Objectives and design

**Fig. 1:**
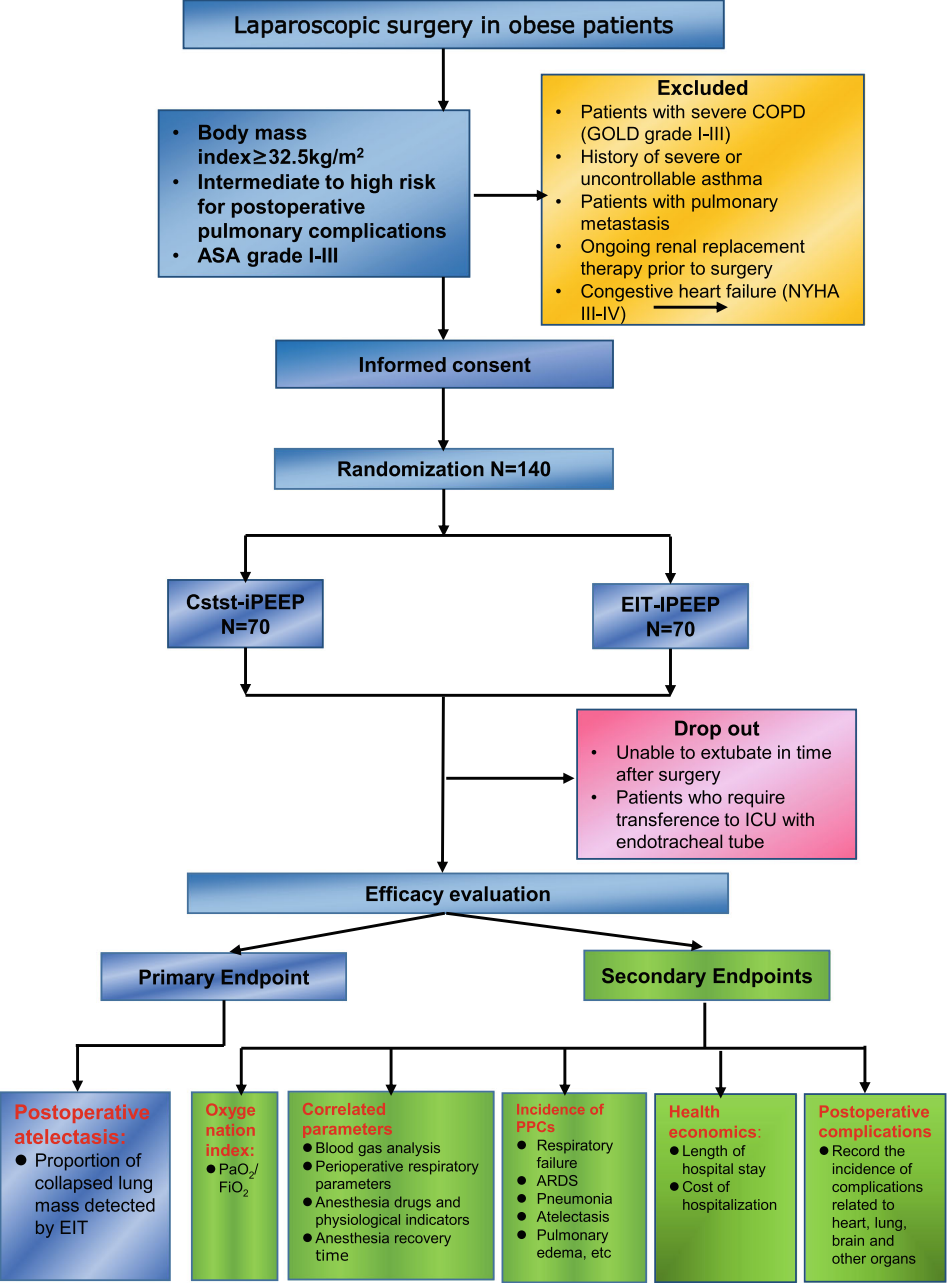
Consolidated Standards of Reporting Trials (CONSORT) diagram for this trial. *PEEP* positive end-expiratory pressure, *iPEEP* individual PEEP, *COPD* chronic obstructive pulmonary disease, *ICU* intensive care unit, *ASA* American Society of Anesthesiologists classification, *PaO*_*2*_ partial pressure of arterial oxygen, *FiO*_*2*_ inspiratory fraction of inspired oxygen, *EIT* electrical impedance tomography, *PPCs* postoperative pulmonary complications

This parallel, two-arm, single-center, prospective, single-blind (investigator-initiated, assessor-blinded), randomized, controlled trial tests the hypothesis that individualized PEEP titration guided by optimal Cstat is a convenient and feasible lung-protective strategy for obese patients at moderate to high risk for PPCs undergoing laparoscopic surgery. 140 patients will be randomly divided into one of two different groups (see Consolidated Standards of Reporting Trials [CONSORT] diagram, Fig. 1).

This trial will be conducted at Beijing Friendship Hospital Affiliated to Capital Medical University. The study conforms to the CIOMS Principles of the International Guidelines for Biomedical Research Involving Human Subjects and the WMA of the Declaration of Helsinki. The study has obtained ethics approval from the Ethics Committee of Beijing Friendship Hospital Affiliated to Capital Medical University. (The approval number is 2020-P2-145-01) and has been registered in the Chinese Clinical Registry (Chictr) (registration number: ChiCTR2000039144).

### Blinding, data collection, randomization, and record-keeping

This is a single-blind randomized clinical trial. Demographic parameters, intraoperative data, postoperative clinical status, fluid balance, hemodynamic variables, respiratory parameters, anesthesia data, laboratory results, and hygienic indicators will be recorded onto the case report forms (CRF). The information of lung collapse calculated by EIT will be stored in the computer and named with an anonymous number.

All participants meeting the inclusion criteria will be randomized to the Cstat-PEEP group or the EIT-PEEP group in a ratio of 1:1. Randomization will be implemented via a computer-generated blocked randomization form, with 35 blocks of four patients per block. The assignment will proceed with opaque, sealed, and numbered envelopes. Subjects shall be enrolled and allocated in numerical order. All original records (CRF and associated correspondence) will be archived and safeguarded for 10 years and then destroyed, as required by hospital standards.

### Study population

Obese patients scheduled for laparoscopic gastric volume reduction surgery under general anesthesia will be screened and recruited during routine preoperative evaluation. Patients are eligible if they meet the following inclusion criteria: 18–60 years old, BMI≥32.5, at the American Society of Anesthesiologists (ASA) Physical Statuses I–III, and at moderate to high risk of developing postoperative pulmonary complications (PPCs). To identify patients at moderate to severe risk for PPCs, the Assess Respiratory Risk in Surgical Patients in Catalonia (ARISCAT) score [9] is applied (see Additional Fig 1). This score predicts individual preoperative risk for developing PPCs with seven predictors, four of which are patient-associated, the other three are surgery-associated. An ARISCAT score ≥26 is related to a moderate to severe risk for PPCs. We use BMI ≥ 32.5 as the standard for morbid obesity under the Asia-Pacific classification [10].

Exclusion criteria are patients aged>60 years or <18 years, ASA grade ≥ IV, with severe chronic obstructive pulmonary disease (COPD, defined as GOLD grades III–IV), having a history of severe or uncontrollable asthma, a history of pulmonary metastasis, in preoperative ongoing renal replacement therapy, with congestive heart failure (defined as NYHA grades III–IV), and failure to be extubated in time after surgery and the need for transference to the ICU with the endotracheal tube.

### Standard procedures

To avoid interfering the trial intervention, components of perioperative anesthesia care (involving general anesthesia, postoperative analgesia, and fluid management) are performed by a fairly fixed team of anesthesiologists in accordance with the clinical routine. The following strategies are recommended:

1. Before surgery, all patients need a thorough assessment of the airway, performed on the basis of 12 predictors of a difficult airway. When more than three of the predictors are present, endotracheal intubation is performed when patients are awake or after slow induction while maintaining spontaneous breathing should be taken into consideration. Adequate equipment, personnel, and medicine required for artificial airway establishment should be prepared in advance (see Additional Table 1: Predictors of Difficult Airway).
2. According to clinical routine, upon admission to the operation room, patients’ following parameters are monitored: electrocardiogram, non-invasive blood pressure, BIS, pulse oximetry, and urine output. Invasive arterial blood pressure is continuously measured after the insertion of an arterial line into the radial or dorsal pedis artery under regional anesthesia.
3. After intravenous and arterial catheterization, an EIT belt will be placed at the fifth intercostal space, and continuous EIT monitoring (PulmoVista 500, Drager) will be started.
4. For patients without predicted difficult airway, anesthesia induction is performed using rapid sequence induction: 0.05mg/kg midazolam is given to patients 15 min before induction; sufentanil, etomidate, rocuronium, or cisatracurium are used for induction. Mechanical ventilation is initiated after endotracheal intubation.
5. Total intravenous anesthesia is applied for the maintenance of anesthesia with propofol and remifentanil.
6. Intraoperative hemodynamics is managed in accordance with surgical procedure and blood loss.
7. Performing postoperative analgesia to ensure a VAS pain score<3. Regional anesthesia or neuraxial block should be applied whenever indicated.
8. Postoperative physiotherapy involving early mobilization and stimulation of cough along with deep breathing exercises should be performed.

Data related to the procedures applied shall be collected in detail and analyzed. Anesthesia care and associated treatment must abide by clinical routines. Intravenous catheters and nasogastric tubes may be used in accordance with guidelines or surgical practice. Urinary bladder catheters are usually not applied routinely for this sort of surgery in our hospital.

### Mechanical ventilation

The following are ventilator settings: We set the driving pressure to 15cmH_2_O, using pressure-control-volume compensation mode (PSV-VC). The lowest possible fraction of inspired oxygen is used (FiO_2_ ≥0.4) to avoid hypoxemia (defined as SpO_2_ >92%). The compensated tidal volume is fixed at 7ml/kg (PBW), and the respiratory rate is set to 12–15 times/minute (adjusted to normocapnia, defined as P_ET_CO_2_ maintained at 35–45 cmH_2_O). Management of anesthesia maintenance is left to the discretion of the anesthesiologist in charge. All complications related to anesthesia are managed in accordance with clinical guidelines.

### Intervention

**Fig. 2:**
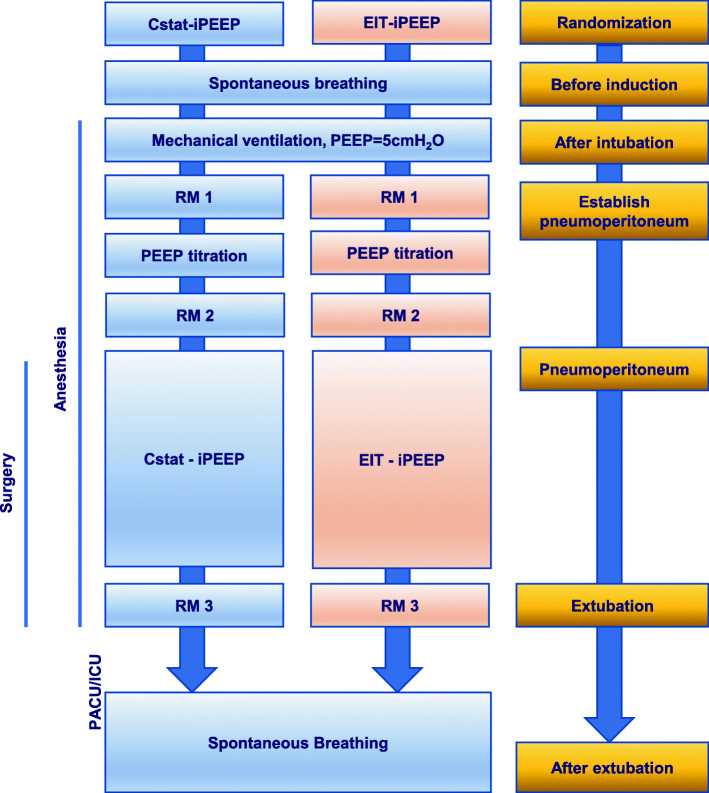
Individualized PEEP and perioperative management process. *iPEEP* individualized positive end-expiratory pressure, *Cstat-iPEEP* optimal static lung compliance-directed iPEEP titration group, *EIT-iPEEP* electrical impedance tomography-guided iPEEP titration group, *RM* the ventilator-driven alveolar recruitment maneuver

After endotracheal intubation, and initiation of mechanical ventilation PEEP is maintained at 5cmH_2_O for 5 minutes, baseline parameters are measured and recorded in all patients during this period. All patients (in both groups) are submitted to ventilator-driven alveolar recruitment maneuvers (RM) 5 min after endotracheal intubation [11], and then, the same procedure is repeated after finishing PEEP titration and before extubation (Fig. 2).

**Fig. 3:**
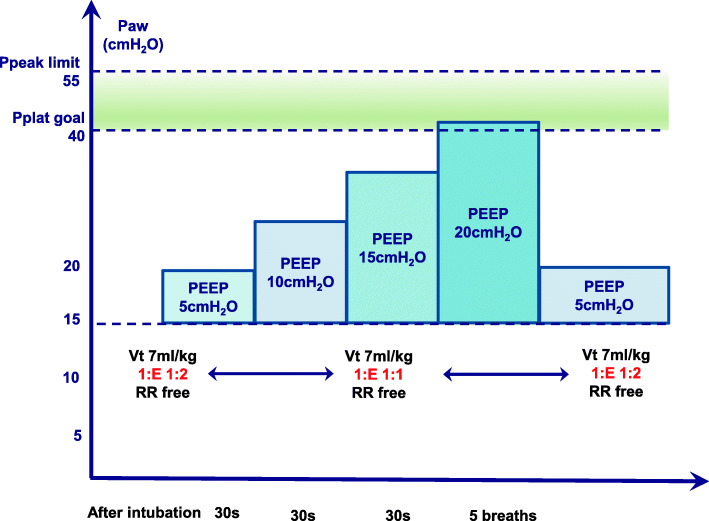
The ventilator-driven alveolar recruitment maneuver protocol. *Ppeak* peak airway pressure. *Pplat* plateau airway pressure. *PEEP* positive end-expiratory pressure, *Vt* tidal volume normalized for adjusted body weight, *I:E* ratio between inspiratory and expiratory time, *RR* respiratory rate

RM is performed in the following steps (Fig. 3):

1. In pressure-controlled ventilation, driving pressure is restrained within 15cmH_2_O.
2. Increase PEEP from 5 to 20 cmH_2_O with 5 cmH_2_O raise each time and keep PEEP constant for 30s after each increase. When PEEP reaches 20 cmH_2_O, resulting in an inspiratory pressure of 40 cmH_2_O, maintain 5 breathing circles at PEEP 20 cmH_2_O until the end.
3. Throughout the duration of the RM, VT is set to 7ml/kg, and I:E is set to 1:1.
4. Ppeak <55 cmH_2_O.
5. All patients receive a standardized fluid regimen along with vasopressors during the RM maneuver according to the protocol to alleviate short-term hemodynamic suppression caused by RM and maintain MAP ≥ 65 mmHg.

**Fig. 4:**
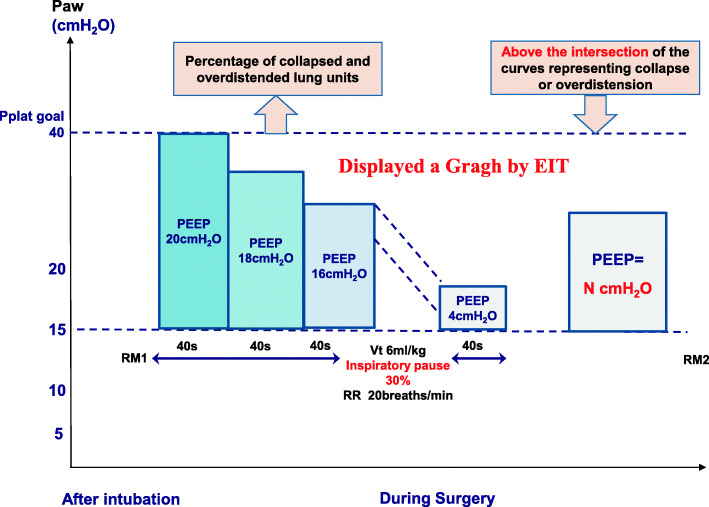
EIT-guided individualized PEEP titration. *Paw* airway pressure, *Pplat* plateau airway pressure, *PEEP* positive end-expiratory pressure, *V*_*T*_ tidal volume normalized for adjusted body weight, *RR* respiratory rate, *RM* the ventilator-driven alveolar recruitment maneuver

After the first RM is performed, the individualized PEEP will be titrated by optimal Cstat or EIT according to their grouping information. According to a study conducted by Pereira SM et al. [12], patients in the EIT-PEEP group will be performed an EIT-guided decremental PEEP titration procedure as following (Fig. 4):

1. After the first RM is performed, immediately after the establishment of pneumoperitoneum, PEEP titration is started. PEEP is started at 20 cmH_2_O and inspiratory pressure at 40 cmH_2_O. PEEP will be decreased at steps of 2 cmH_2_O every 40s, and respiratory rate maintained at 20 breaths/min, inspiratory pause at 30%, and *V*_*T*_ = 6 ml/kg.
2. After the procedure comes to an end, a graph is automatically plotted and displayed by the EIT monitor, which shows the percentage of collapsed and overdistended lung units (equivalent to the percent mass of overdistended or collapsed lung tissue) at each PEEP level.
3. PEEP-EIT is determined as the nearest PEEP above the intersection of the curves representing collapse or overdistension, which indicates a mechanical compromise minimizing both overdistension and collapse of the lung tissue.

**Fig. 5:**
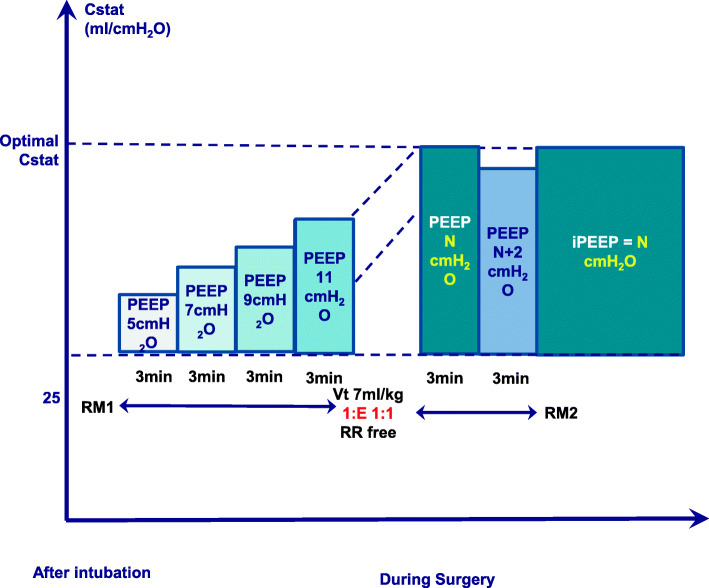
Individualized PEEP titrated by optimal Cstat. *Cstat* static lung compliance, *PEEP* positive end-expiratory pressure, *iPEEP* individualized positive end-expiratory pressure, *RM* the ventilator-driven alveolar recruitment maneuver

In the Cstat-iPEEP group, patients’ iPEEP is obtained through the following procedure (Fig. 5):

1. The first RM (RM1) is performed 5 min after intubation.
2. Limit the peak airway pressure to lower than 55 cmH_2_O.
3. *V*_*T*_ is fixed to 7ml/kg (predicted body weight, PBW), I: E to 1:1, respiratory rate to 12–15 breaths/min.
4. Titration procedure: Immediately after the establishment of pneumoperitoneum, PEEP titration is started. Initial PEEP is set to 5 cmH_2_O. PEEP is increased at steps of 2 cmH_2_O, and each PEEP level is sustained for 3 min, during which Cstat is calculated (in accordance with the formula: Cstat = V_T_ /Plat-PEEP). We end the titration process the moment the calculated Cstat displays a downward trend and choose its previous PEEP (the PEEP that maximizes Cstat) as the optimal PEEP for this patient.
5. The upper limit of PEEP is set to 20 cmH_2_O.
6. After the ideal PEEP is obtained, we perform the second RM2.
7. The third RM3 is performed before extubation.

### Study endpoints

The primary endpoint of the study is the amount of calculated atelectasis, corresponding to the percentage of collapsed lung mass, which is obtained through the following steps. The baseline lung volume and its associated parameters of all patients are measured before and after anesthesia induction by Drager’s EIT. Immediately after extubation and 2 h postoperation, the proportion taken up by collapsed lung mass is measured by EIT. The amount of atelectasis is calculated via dividing the area occupied by non-ventilated lung mass by baseline lung volume.

Secondary endpoints consist of the value of individualized PEEP, oxygenation index (PaO_2_/FiO_2_), respiratory parameters, blood gas analysis indicators, anesthesia-associated parameters, and hygienic index.

1. Oxygenation index: At before intubation (T0), after the third RM and before extubation (T1), arterial blood is drawn, blood gas analysis is performed, and oxygenation index is calculated.
2. Respiratory parameters: Every 5 min, RR, VT, Ppeak, Pplat, and PEEP are recorded.
3. Blood gas analysis indicators: At before intubation (T0), after the third RM and before extubation (T1), arterial blood is drawn and blood gas analysis is performed.
4. Anesthesia-associated parameters: hemodynamic parameters, recovery time, anesthetic dosage, and occurrence of hypoxemia are recorded constantly.
5. Tertiary outcomes: length of ICU stay, length of hospital stay, costs of treatment, postoperative complications, and other adverse events are recorded.
6. We define PPCs as follows:
7. Mild respiratory failure: PaO_2_/FiO_2_ < 300 or SpO_2_<90%, measured after at least 10 min of breathing room air, but could be alleviated by inhaling supplementary oxygen of 2L/min, hypoventilation excluded.
8. Moderate respiratory failure: PaO_2_/FiO_2_ < 300 or SpO_2_<90%, measured after at least 10 min of breathing room air, but could be alleviated only by inhaling supplementary oxygen>2L/min, hypoventilation excluded.
9. Severe respiratory failure: requiring noninvasive or invasive mechanical ventilation, excluding hypoventilation due to the use of narcotics.
10. ARDS (according to Berlin definition).
11. Bronchospasm (newly detected expiratory wheezing, responding to bronchodilators).
12. New pulmonary infiltrates (new monolateral or bilateral infiltrates demonstrated by chest X-ray, without other clinical signs.)
13. Pulmonary infection (new or progressive radiographic infiltrate plus at least two of the following: requirement of antibiotic treatment, tympanic temperature >38°C, leukocytosis, or leukopenia [white blood cell count <4000 cells/mm^3^ or > 12,000 cells/mm^3^], and/or purulent sputum).
14. Aspiration pneumonitis (respiratory failure resulting from inhalation of regurgitated gastric contents).
15. Pleural effusion (one of the following demonstrated by chest X-ray: blunting of the costophrenic angle, loss of the sharp silhouette of the ipsilateral hemidiaphragm in the upright position, evidence of adjacent anatomical structures displacement, or [in supine position] a hazy opacity in one hemithorax with preserved vascular shadows).
16. Atelectasis (lung opacities with a shift of the hilum, mediastinum, or hemidiaphragm toward the affected area, along with compensatory overdistension in the adjacent nonatelectatic lung).
17. Cardiopulmonary edema (clinical signs of congestion, including dyspnea, edema, rales, and jugular venous distention, with chest X-ray demonstrating an increase in vascular markings and diffuse alveolar interstitial infiltrates).
18. Pneumothorax (air in the pleural cavity, without vascular bed around the visceral pleura).

Patients conforming to one of the above conditions are deemed as positive for PPCs.

### Study visits and data collection

We visit patients preoperatively, intraoperatively, and postoperatively on postoperative days 1 and 3 and at discharge (Table 1). At all the above-listed points in time, patients’ data are collected and recorded.

1. Preoperative indicators: gender, age, weight, height, body mass index, smoking status, assessment of difficult airways, ASA grade, heart rate, blood pressure, body temperature, blood routine, blood biochemistry, coagulation function, and other past histories.
2. Intraoperative indicators: heart rate, blood pressure, pulse oxygen saturation, bispectral index, respiratory parameters, PEEP settings, all data measured by EIT, transfusion of blood products, volume of fluids and dosage of drugs administered throughout the duration of anesthesia (e.g., crystalloid fluid, colloid fluid, analgesic drugs, sedative drugs, muscle relaxants, vasoactive drugs), urine output, duration of operation (from incision to closure), etc.
3. Postoperative indicators: duration of ICU treatment, length of hospital stay, hospitalization expenses, presence of complications, and adverse events.

**Table 1:**
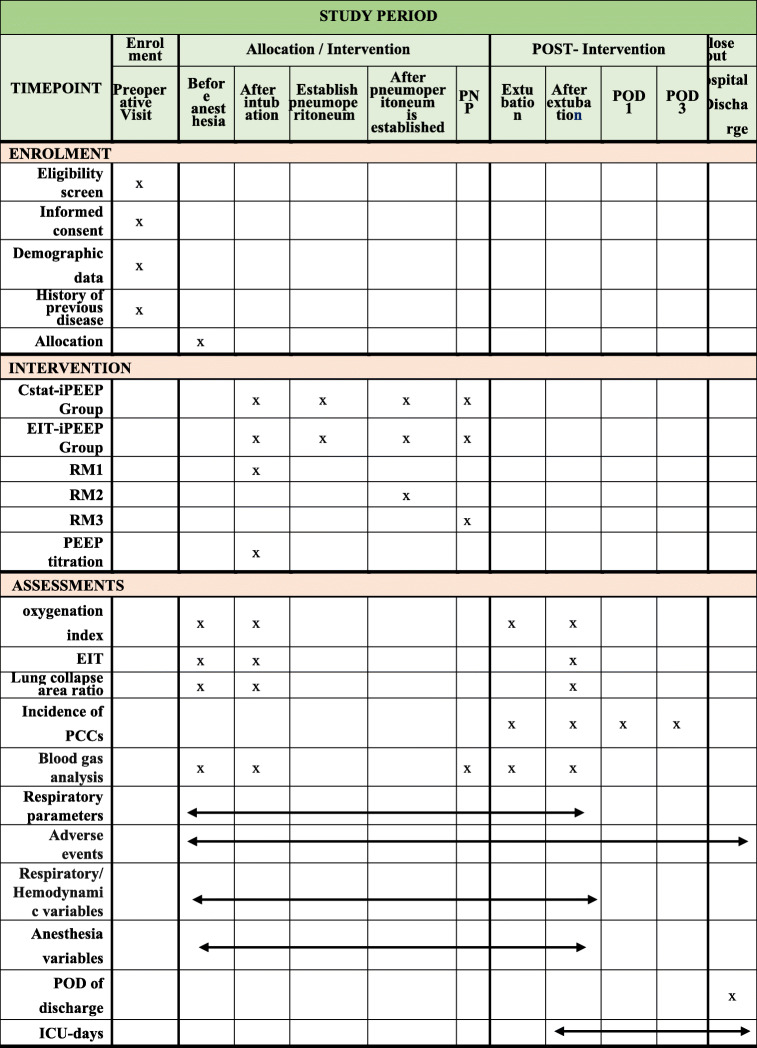
Standard protocol items: time schedule of enrollment, interventions, and assessments

*PNP* pneumoperitoneum period, *POD1* postoperative day 1, *POD3* postoperative day 3, *PEEP* positive end-expiratory airway pressure, iPEEP individual PEEP, *RM* recruitment maneuver, *EIT* electrical impedance tomography, *PNP* pneumoperitoneum, *PPCs* postoperative pulmonary complications, *POD* postoperative day, *ICU* intensive care unit

### Study dropouts

As participation in the study is voluntary, all subjects are free to withdraw their consent to participate in the trial at any time and for any reason without any further treatment or any consequences. Moreover, participation of any subjects could be terminated by an investigator at any time, if he or she believes it is in the best interest of the subject. We shall document the reasons and circumstances of the study discontinuation in the CRF.

### Sample size calculations

The primary endpoint of this study is the ratio of collapsed lung mass to normal lung tissue, which is measured and automatically calculated via using EIT before anesthesia and immediately after endotracheal extubation. According to our pre-experimental results of this study, the lung collapse area in the Cstat-PEEP group was 6.5%, while that in the EIT-PEEP group was 6.2%. We apply a noninferiority test. We hypothesize that compared with the ratio of lung collapse area in the EIT-PEEP group, the ratio of lung collapse area in the Cstat-PEEP group will increase by no more than 2% (i.e., the ratio of lung collapse area in Cstat-PEEP group within 8.2% is non-inferior). We choose a two-sided significance level of 0.025 (*α*) and *β*= 0.2, with a power of 80%. Using the PASS 11.0 software, a sample size of 64 is calculated for each group. Taking a 10% dropout rate in patients’ follow-up into account, a final sample size of 70 is required for each group.

### Data monitoring

The trial is organized by a team comprised of a principal investigator, a general investigator, and other participants who take part in the design and implementation of the study. For quality control purposes, an external independent physician not involved in the trial will be responsible for data monitoring. Monitoring includes assessment of the progress of the study and verification of accuracy as well as the completeness of recorded data. After completion of the study, we will submit the original data and results to the scientific management committee, and after the publication of results, we will disclose original data and results to the public.

### Statistical analysis

After completing the trial, the research team will collaborate with medical statisticians to analyze the data. Statistical analysis will be performed on the basis of intention to treat. SPSS 20.0 software will be used for analysis. The majority of data will be collected and recorded onto the CRF. Before analyzing the data, the pattern of missing data will be evaluated. Analyzation of graphics and data acquired from the EIT machine will be executed by a computer expert familiar with machine principles. Kolmogorov-Smirnov analysis will be used to detect the distribution of all the data. Normally distributed data will be expressed by the mean and standard deviation (SD), while skewed data will be expressed by their median and interquartile range. Unpaired *t* test will be used for univariate analysis of normally distributed data. Pearson correlation test will be employed to test the correlation between two variables. Mann-Whitney *U* test and Wilcoxon signed-rank test will be applied for skewed data. Categorical variables will be assessed by Fisher’s exact test, chi-square tests, or relative risk, if appropriate. 95% CI will be employed to express statistical uncertainty. *P* value <0.05 shall be regarded as significant.

## Discussion

This trial is sufficiently powered to test the hypothesis that an individualized PEEP titrated by maximizing Cstat is not inferior to individualized PEEP determined by EIT in terms of lowering the proportion of collapsed lung tissue, for obese patients. The result will prove that the method of best Cstat titration iPEEP is simple and effective in clinical practice.

According to a population-based study, in 2014, approximately 10.8% of men and 14.9% of women are obese globally [13]. In obese patients, the respiratory compliance is further limited by the cephalic movement of the diaphragm. In addition, the FRC and alveolar ventilation volume of obese patients are much lower than their non-obese counterparts. The incidence of atelectasis in obese patients under general anesthesia is as high as 90% [14]. In obese patients under general anesthesia and mechanical ventilation, the proportion of normal ventilated lung tissue drops from 71 to 50%, while the proportion of insufficiently ventilated lung mass increases from 28 to 39%, and the proportion of non-ventilated lung tissue elevates from 1 to 11%. In sharp contrast, the proportion of atelectatic lung mass is only 3% in normal weight patients under general anesthesia and mechanical ventilation [15].

Because airway pressure required to keep lung units from collapsing is much lower than the pressure needed to recruit atelectatic lung units, recruitment maneuver could significantly improve ventilation. Applying PEEP after a successful recruitment maneuver could prevent the rapid reformation of atelectasis, especially when the fraction of inspired oxygen is as high as 100% [16]. Applying PEEP alone alleviates the reduction of end-expiratory lung volume and lung elastance imposed by pneumoperitoneum on obese patients, but fails to reduce an atelectatic fraction of the lung tissue [17]. Compared to PEEP applied alone, the combination of PEEP and recruitment maneuver leads to greater enhancement in lung elastance and results in lower airway pressure as well as resistance in obese patients undergoing laparoscopic surgery.

However, no agreement has been reached concerning the optimal PEEP for obese patients. High PEEP is associated with improved intraoperative oxygenation and lung elastance as well as a reduced proportion of atelectatic lung units, especially for obese patients [18]. But too high PEEP may lead to overextension of lung units, which aggravate lung injury. From the PROBESE study, compared to a lower PEEP, a fixed higher PEEP did not decrease the incidence of PPCs in obese patients [1].

According to the literature, it is highly unlikely to find a fixed PEEP which suits all patients with significant variation in PEEP requirement caused by individual characteristics, such as abdominal content, chest wall shape and dimensions, pleural pressures, and lung weights [19]. Thus, an individualized ideal PEEP may be superior to a fixed PEEP level. Relevant studies have discovered that compared with applying a fixed PEEP, applying individualized PEEP improves oxygenation and respiratory compliance, reduces the fraction of dead space, minimizes atelectasis, optimizes ventilation distribution, and ameliorates lung injury by lowering driving pressure [20, 21].

No consensus has been reached concerning the ideal way to select the ideal and individualized PEEP. And, many ways effective for selecting individualized PEEP for normal weight patients may not be appropriate for obese patients. For instance, determining individualized PEEP as the pressure nearest to the low inflection point of the pressure-volume curve underestimates the PEEP required by obese patients [22]. Electrical impedance tomography (EIT) is deemed superior to traditional ways of individualized PEEP titration by many studies [23, 24]. EIT is a clinical imaging tool that monitors local ventilation distribution. It enables the continuous measurement of local lung ventilation changes resulting from the application of recruitment maneuvers and PEEP as well as adjustments in tidal volume and fraction of inspired oxygen. Traditional ways of PEEP titration may provide misleading information by averaging contradicting pathological characteristics of different lung units, such as overdistention and atelectasis. In contrast, EIT monitors regional lung ventilation and can detect pulmonary heterogeneity in a dynamic process. It has gained widespread interest in guiding lung-protective ventilation in obese patients. However, the EIT instrument is unaffordable by many clinical institutions, which limits its use in routine clinical practice. At the same time, the bandage of the EIT machine conflicts with the field disinfection of many operations in the chest or abdomen, which also limits its application in the perioperative period. So alternative ways to titrate PEEP both affordable and effective need to be found out. This study is going to test the efficacy of determining individualized PEEP during an incremental titration procedure guided by optimal static lung compliance by comparing it with the PEEP titration strategy directed by EIT.

In our previous study [25], it has been found that when applying optimal static lung compliance titration iPEEP in obese patients undergoing laparoscopic surgery, the PEEP value was about 10.5 cmH_2_O. Compared with PEEP 5 cmH_2_O, it could significantly improve the oxygen index and respiratory function of obese patients during and after laparoscopic surgery, combined with other lung protective strategies. These data provide evidential support for us to use the best static lung compliance method to titrate iPEEP.

In conclusion, this study aims to verify the following hypothesis: individualized iPEEP titrated to optimal static lung compliance is not inferior to individualized iPEEP obtained during a titration procedure guided by EIT, in terms of improving intraoperative oxygenation and ameliorating postoperative atelectasis in obese patients undergoing laparoscopic surgery. This study will provide a convenient and feasible strategy for individualized PEEP titration in obese patients. The result shall provide direct evidence to further refine lung-protective ventilation strategies in obese patients and exploit the significance of lung-protective ventilation in ERAS in obese patients.

### Trial status

We enrolled the first participant of the trial on November 15, 2020, the protocol version is the first version, and the version number is V1.0/2020.06.26. We will complete the recruitment on September 17, 2022. This trial is still ongoing.

## Supplementary Information

**Additional file 1: Additional Fig 1**. Independent predictors of risk for development of postoperative pulmonary complications as described by Canet et al .[1] (ARISCAT score). **Additional Table 1**. Difficult Mask Ventilation Combined with Difficult Laryngoscopy Prediction Scor e[2].

## Data Availability

Data will be disclosed to the public after the complication of the trial. After the study is completed and the results are published, the data will be obtained by contacting the corresponding author.

## Abbreviations

PPCs: Postoperative pulmonary complications
iPEEP: Individualized positive end-expiratory pressure
Cstat: Static lung compliance
FiO2: Inspiratory fraction of inspired oxygen
EIT: Electrical impedance tomography
PBW: Predicted body weight
RM: Recruitment maneuvers
VT: Tidal volume
CRF: Case report forms
ARISCAT: The assess respiratory risk in surgical patients in catalonia
BMI: Body mass index
COPD: Chronic obstructive pulmonary disease
Cstat-iPEEP: Optimal static lung compliance-directed iPEEP titration group
EIT-iPEEP: Electrical impedance tomography-guided iPEEP titration group
VAS: Visual analog scale
PSC-VC: Pressure-control-volume compensation mode
Pplat: Plateau airway pressure
Ppeak: Peak airway pressure
1:E: Ratio between inspiratory and expiratory time
PaO_2_: Partial pressure of arterial oxygen
PCT: Procalcitonin
