# Supplementary material for: Effects of individualized PEEP obtained by two different titration methods on postoperative atelectasis in obese patients: study protocol for a randomized controlled trial
Source: Trials. 2021 Oct 15;22:704. doi: 10.1186/s13063-021-05671-1 (PMC8517565; doi:10.1186/s13063-021-05671-1)
Supplement: Supplementary file 1 — Additional file 1: Additional Fig 1. Independent predictors of risk for development of postoperative pulmonary complications as described by Canet et al .[1] (ARISCAT score). Additional Table 1. Difficult Mask Ventilation Combined with Difficult Laryngoscopy Prediction Scor e[2]. [file 13063_2021_5671_MOESM1_ESM.docx]

**Additional File 1:**

Additional Fig 1.


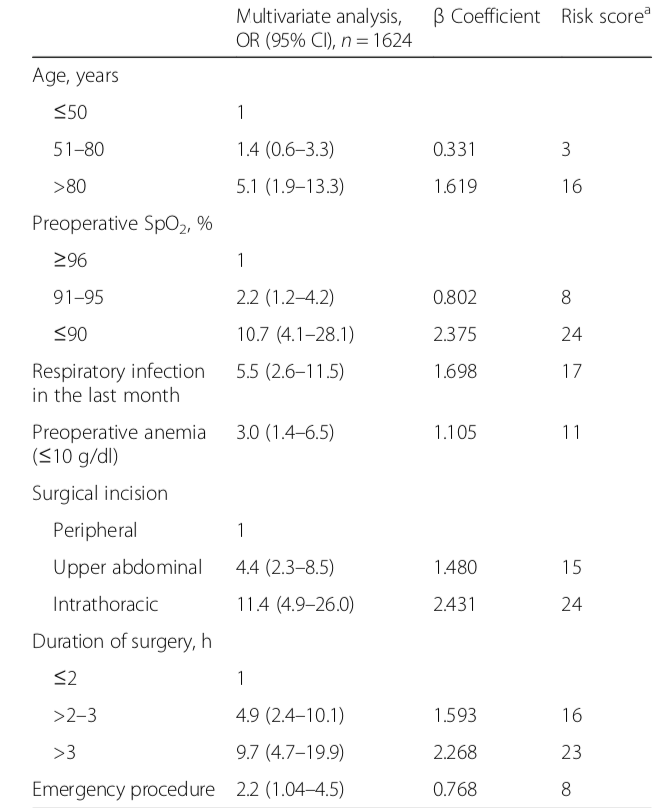


**Fig 1.** Independent predictors of risk for development of postoperative pulmonary complications as described by Canet et al. **^[1]^** (ARISCAT score). A risk score ≥26 predicts an intermediate to high risk for postoperative pulmonary complications).

^a^ The simplified risk score is the sum of each logistic regression coefficient multiplied by 10, after rounding off its value.

Additional table 1. Difficult Mask Ventilation Combined with Difficult Laryngoscopy Prediction Score.^[2]^


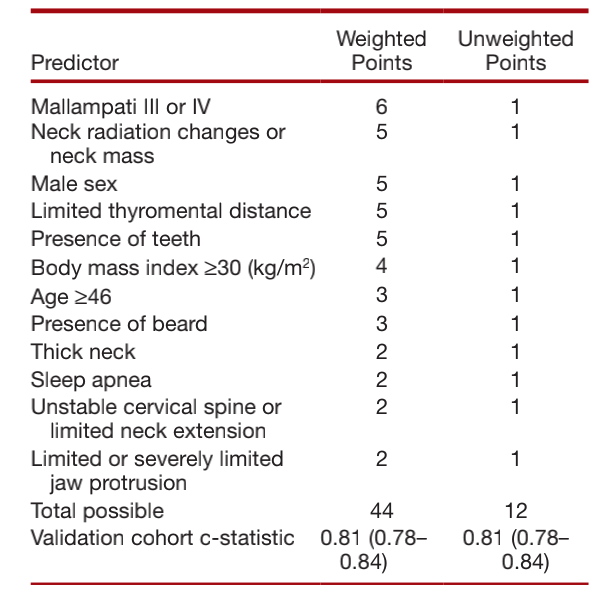


**Reference:**

1. Bluth T, Teichmann R, Kiss T, et al. Protective intraoperative ventilation with higher [versus lower levels of positive end-expiratory pressure in obese patients (PROBESE): study protocol for a randomized controlled trial.](https://www.ncbi.nlm.nih.gov/pubmed/28454590) Trials. 2017; 18(1): 202-24.
2. [Kheterpal S](https://www.ncbi.nlm.nih.gov/pubmed/?term=Kheterpal%20S%5BAuthor%5D&cauthor=true&cauthor_uid=24071617), [Healy D](https://www.ncbi.nlm.nih.gov/pubmed/?term=Healy%20D%5BAuthor%5D&cauthor=true&cauthor_uid=24071617), [Aziz MF](https://www.ncbi.nlm.nih.gov/pubmed/?term=Aziz%20MF%5BAuthor%5D&cauthor=true&cauthor_uid=24071617), et al. Incidence, predictors, and outcome of difficult mask ventilation combined with difficult laryngoscopy: a report from the multicenter perioperative outcomes group. [Anesthesiology.](https://www.ncbi.nlm.nih.gov/pubmed/?term=Anesthesiol.+2013%3B+119%3A+1360-9) 2013 Dec;119(6):1360-9.
